# The coupling of leaf, litter, and soil nutrients in warm temperate forests in northwestern China

**DOI:** 10.1038/s41598-017-12199-5

**Published:** 2017-09-18

**Authors:** Guangqi Zhang, Ping Zhang, Shouzhang Peng, Yunming Chen, Yang Cao

**Affiliations:** 1 College of Forestry, Northwest A&F University, Yangling, 71210 Shaanxi China; 2 State Key Laboratory of Soil Erosion and Dry land Farming on Loess Plateau, Northwest A&F University, Yangling, 712100 Shaanxi China; 3 Institute of Soil and Water Conservation, Chinese Academy of Sciences and Ministry of Water Resources, Yangling, 712100 Shaanxi China

## Abstract

The nutrient ecological stoichiometry of plants and soil is important for the growth and dynamics of species, but the stoichiometric relationships among leaf, litter, and soil remain poorly understood. We analyzed the carbon (C), nitrogen (N), and phosphorus (P) stoichiometry of the leaves, litter, and soil for 31 species at 140 sites in warm temperate forests in northwestern China to document the patterns of nutrient traits and their relationships with climatic factors. The average concentrations of C, N, and P in the combined forests were 462.97, 18.04, and 1.32 g kg^−1^ for leaves, 365.12, 12.34, and 0.87 g kg^−1^ for litter, and 15.72, 1.29, and 0.54 g kg^−1^ for soil, respectively. The concentrations differed significantly among the leaves, litter, and soil. Leaf and soil nutrients were not significantly correlated, whereas leaf and litter nutrients and litter and soil nutrients were significantly correlated, indicating that litter provided a link between leaves and soil and demonstrating the nutrient associations among leaves, litter, and soil. Soil nutrients were strongly correlated with climatic factors, and precipitation had a larger impact than temperature on the plants and soil. This study will help to predict the growth and dynamics of species under environmental changes.

## Introduction

Ecological stoichiometry, which balances energy and multiple chemical elements and unifies the theories of various ecological disciplines, has greatly advanced our understanding of ecological dynamics and processes^1–3^. Carbon (C), nitrogen (N), and phosphorus (P), the three most basic elements in organisms, play crucial roles in the activities of organisms^4^. N and P, the main elements limiting plant growth, affect plant productivity^5^, the photosynthetic rate^6^ and other ecosystem functions^7^. The critical ratios of soil nutrients are also indicators of nutrient supply for plant growth^8–11^.

Terrestrial ecosystems are more complex than marine ecosystems owing to the various conditions (e.g., topography, vegetation, human intervention, etc.), and C, N, and P are biologically coupled by their influences on the biochemical reactions that control photosynthesis, respiration and decomposition in terrestrial ecosystems^2,12,13^. As integral parts of terrestrial ecosystems, leaves, litter, and soil play important roles in ecosystem development, succession, and elemental cycling. Soil is the substrate in which vegetation grows, and it provides most of the nutrients required for growth. The capacity and intensity of the supply of soil nutrients influence the C, N, and P contents in different plant organs. In general, as the key process and main repository of the nutrient cycle and energy flow in ecosystems, the leaf-litter-soil system plays an important role in the nutrient cycling of terrestrial ecosystems^14^. Litter is the basic carrier of nutrients from plants to soil. This dynamic exchange can achieve and maintain a balance between soil nutrients and the elemental ratios required for plant growth^15^. The ability to store carbon and the rate of accumulation and supply of N and P that limit plant growth are closely associated^16^. The stoichiometries of C, N, and P are powerful tools for determining their coupling mechanisms and nutrient limitations in terrestrial ecosystems, and numerous studies have reported the individual stoichiometries for leaves, litter, and soil^11,17–20^. However, the potential nutrient cycles of the C, N, and P stoichiometries in the leaf-litter-soil system are far from complete, and the coupling relationships among leaf, litter, and soil remain poorly understood.

Several factors have been proposed to explain the patterns of plant and soil chemical traits. Among these, climate and plant growth form are thought to be the primary factors. The effect of environmental factors on plant and soil stoichiometry on a regional scale has become a popular field of study. A number of studies have been conducted to explore the geographical pattern of nutrient elements along environmental gradients^11,19,21–25^. These studies have analyzed nutrients in various plant components, such as green leaves^22,23^, senesced leaves^24^, and soil^19^, but the results have been inconsistent. For example, Reich and Oleksyn^22^ found that leaf N and P concentrations decreased but N: P increased globally in response to climatic factors (e.g., increases in mean annual temperature (MAT) and mean annual precipitation (MAP)). In contrast, Wright *et al*.^23^ found that the modification of leaf traits by climate was surprisingly modest globally. In addition, Han *et al*.^17^ found differences in the distributions of N and P between plants in China and globally. Furthermore, Yuan and Chen^24^ found that the senesced-leaf N concentration significantly increased but the P concentration decreased as MAT and MAP increased, whereas Chen *et al*.^19^ found that the soil N and P concentrations both decreased significantly in response to climatic factors. These results contribute to our understanding of the dynamics of plant and soil elements under large environmental perturbations, but little is known about the differences in the responses of leaves, litter, and soil nutrients to climatic factors and plant types, especially on the Loess Plateau in China. Are the relationships between leaf, litter, and soil nutrients and climatic factors consistent? How are the leaf, litter, and soil nutrients affected by climatic factors? What are the relationships among leaf, litter, and soil nutrients? Studying the relationships of leaf, litter, and soil nutrients with climatic factors (e.g., temperature and precipitation) simultaneously is important to help to resolve these questions and to provide practical references for understanding the role of nutrient cycles in forest growth across broad geographic scales and their dynamics under global warming.

Therefore, the objective of this study was to determine the ecological stoichiometries of leaf, litter, and soil nutrients of warm temperate forests and their relationships with climatic factors in northwestern China. Specifically, we (i) compared the concentrations and ratios of the leaf, litter, and soil nutrients of different plant types on the Loess Plateau, (ii) quantified the correlations among the leaf, litter, and soil nutrients, and (iii) determined the relationships of the spatial variations of leaf, litter, and soil nutrients with climatic factors.

## Results

### Characteristics and ecological stoichiometry of leaf, litter, and soil nutrients

The average C, N, and P concentrations for all 420 samples were 462.97, 18.04, and 1.32 g kg^−1^ for leaves, 365.12, 12.34, and 0.87 g kg^−1^ for litter, and 15.72, 1.29, and 0.54 g kg^−1^ for soil, respectively (Table 1). The nutrient concentrations differed significantly between leaves, litter, and soil (*P* < 0.05). The C, N, and P concentrations were higher in the leaves than in the litter and soil, whereas C: N, N: P, and C: P were higher in the litter than in the leaves and soil. The leaf, litter, and soil P concentrations were the most variable, with overall mean coefficients of variation (CVs) of 0.44, 0.31, and 0.66, followed by C (CV = 0.08, 0.16, and 0.55) and N (CV = 0.43, 0.29, and 0.52) (Supplementary Table S1). The leaf and litter C concentrations and C: N and C: P were lower in broadleaved forests than in coniferous forests (*P* < 0.05) but did not differ significantly between natural forest and plantation. Only the soil N concentration and C: N differed significantly between broadleaved and coniferous forests, with a higher N concentration and lower C: N in broadleaved forests (*P* < 0.05). The soil C and N concentrations and N: P and C: P differed significantly between natural forest and plantation and were higher in natural forest (*P* < 0.05) (Table 1).

**Table 1:** Concentrations (g kg^−1^) and ratios of plant and soil nutrients (mean ± SE).

Component	Plant type/origin	C	N	P	C:N	N:P	C:P
Leaf	Broadleaf	451.60 ± 2.90b	20.23 ± 0.73a	1.42 ± 0.06a	20.06 ± 1.37b	15.19 ± 0.45a	365.59 ± 13.89b
	Conifer	501.06 ± 4.18a	10.65 ± 0.49b	0.96 ± 0.05b	51.60 ± 3.38a	11.63 ± 0.54b	566.92 ± 28.63a
	Natural forest	461.70 ± 3.49a	16.70 ± 0.45b	1.24 ± 0.06a	31.03 ± 1.79a	14.54 ± 0.51a	422.86 ± 16.68a
	Plantation	464.57 ± 5.20a	19.73 ± 1.37a	1.41 ± 0.07a	32.98 ± 2.81a	14.17 ± 0.60a	397.45 ± 24.94a
	All	462.97 ± 3.01A	18.04 ± 0.67A	1.32 ± 0.05A	31.89 ± 1.59A	14.38 ± 0.39B	411.61 ± 14.42B
Litter	Broadleaf	351.70 ± 5.22b	13.10 ± 0.34a	0.92 ± 0.02a	28.70 ± 0.72b	15.28 ± 0.41a	423.84 ± 13.15b
	Conifer	410.42 ± 7.67a	9.77 ± 0.42b	0.70 ± 0.05b	44.73 ± 1.80a	16.02 ± 0.99a	737.83 ± 70.86a
	Natural forest	365.71 ± 5.44a	11.92 ± 0.25a	0.85 ± 0.03a	32.02 ± 0.75a	15.21 ± 0.45a	475.58 ± 15.59a
	Plantation	364.38 ± 8.61a	12.86 ± 0.61a	0.89 ± 0.04a	32.79 ± 1.80a	15.75 ± 0.66a	520.80 ± 45.66a
	All	365.12 ± 4.85B	12.34 ± 0.30B	0.87 ± 0.02B	32.36 ± 0.90A	15.45 ± 0.38A	495.61 ± 21.99A
Soil	Broadleaf	15.81 ± 0.84a	1.35 ± 0.07a	0.57 ± 0.04a	11.86 ± 0.25b	2.84 ± 0.14a	33.51 ± 1.77a
	Conifer	15.43 ± 1.48a	1.08 ± 0.08b	0.44 ± 0.03a	14.23 ± 0.62a	2.89 ± 0.23a	40.45 ± 3.46a
	Natural forest	17.20 ± 0.98a	1.45 ± 0.08a	0.54 ± 0.05a	11.98 ± 0.27a	3.39 ± 0.16a	40.78 ± 2.17a
	Plantation	13.86 ± 1.05b	1.09 ± 0.08b	0.56 ± 0.03a	12.94 ± 0.45a	2.17 ± 0.14b	27.95 ± 2.00b
	All	15.72 ± 0.73C	1.29 ± 0.06C	0.54 ± 0.03C	12.40 ± 0.25B	2.85 ± 0.12C	35.10 ± 1.59C

Different lowercase letters indicate significant differences between plant forms. Different uppercase letters indicate significant differences between components (*P* < 0.05).

### Correlations among the leaf, litter, and soil nutrients

The leaf and litter nutrients and the litter and soil nutrients were strongly correlated for the combined forests (*P* < 0.01), but the leaf and soil nutrient concentrations and ratios were not significantly correlated, except for C: P (Table 2). The traits of the leaf and litter nutrients were significantly correlated in broadleaved forests (*P* < 0.01) but not in coniferous forests, except for N: P and C: P. The leaf and soil N concentration and C: P were correlated (*P* < 0.05), and the litter and soil nutrients and their ratios were correlated (*P* < 0.01), except for the N concentration and C: N in broadleaved forests. The traits of the leaf and soil nutrients and the litter and soil nutrients were not correlated in the coniferous forests. Only the N concentration and N: P were not significantly correlated in natural forest, and the other leaf and litter nutrient concentrations and ratios were significantly correlated in both natural forest and plantation. Only leaf and soil C: P were significantly correlated in both natural forest and plantation (*P* < 0.05). Although the litter and soil C concentrations and C: N in natural forest and the N concentration and C: N in plantation were not correlated, there were strong correlations (*P* <0.01) between the litter and soil nutrients and their ratios (Table 2).

**Table 2:** Correlations among leaf, litter, and soil C, N, and P concentrations and ratios (Pearson test).

Component	Plant type/origin	C	N	P	C:N	N:P	C:P
Leaf-litter	Broadleaf	0.252^**^	0.587^**^	0.499^**^	0.380^**^	0.344^**^	0.473^**^
	Conifer	−0.045	0.181	0.169	0.198	0.385^*^	0.617^**^
	Natural forest	0.274^*^	0.117	0.312^**^	0.303^**^	0.213	0.357^**^
	Plantation	0.494^**^	0.743^**^	0.702^**^	0.689^**^	0.385^**^	0.762^**^
	All	0.404^**^	0.638^**^	0.505^**^	0.565^**^	0.297^**^	0.616^**^
Leaf-soil	Broadleaf	0.030	−0.232^*^	0.098	−0.062	−0.012	0.357^**^
	Conifer	−0.242	0.006	0.148	−0.064	0.084	0.091
	Natural forest	−0.094	0.073	0.134	0.184	0.071	0.266^*^
	Plantation	0.057	−0.119	0.210	0.106	−0.163	0.402^**^
	All	−0.026	−0.096	−0.146	0.142	−0.0003	0.325^**^
Litter-soil	Broadleaf	0.349^**^	0.140	0.488^**^	−0.146	0.331^**^	0.580^**^
	Conifer	0.034	0.039	0.149	0.306	0.231	0.258
	Natural forest	0.184	0.401^**^	0.538^**^	0.151	0.292^**^	0.417^**^
	Plantation	0.336^**^	0.150	0.392^**^	0.246	0.473^**^	0.601^**^
	All	0.255^**^	0.183^**^	0.453^**^	0.219^**^	0.301^**^	0.400^**^

^*^ *P* < 0.05, **P < 0.01.

### Variations of leaf, litter, and soil nutrients and their ratios with climatic factors

The leaf and litter N and P concentrations and N: P were weakly correlated with the climatic factors for the combined forests. The leaf N concentration was correlated with MAP (*P* < 0.05), and the leaf N: P was correlated with MAP and mean annual extreme high temperature (MAEHT) (*P* < 0.01). The soil N and P concentrations and N: P were strongly correlated with climatic factors (especially MAP), but the soil N: P and MAT, soil N concentration and mean temperature in August (MT_max_), soil N concentration and mean temperature in January (MT_min_), and soil P concentration and MAEHT were not significantly correlated (Table 3).

**Table 3:** Correlations between climatic factors and leaf, litter, and soil N and P concentrations and ratios (Pearson test).

Component	Element	MAP	MAT	MT_max_	MT_min_	MAEHT	MAELT
Leaf	Ln N	−0.198^*^	−0.037	0.025	−0.133	0.056	−0.146
	Ln P	0.024	−0.092	−0.098	−0.057	−0.121	−0.083
	Ln N:P	−0.248^**^	0.047	0.123	−0.097	0.180^*^	−0.086
Litter	Ln N	0.090	−0.112	−0.138	−0.019	−0.184	−0.057
	Ln P	0.082	−0.007	−0.018	0.001	−0.020	−0.005
	Ln N:P	−0.017	−0.067	−0.076	−0.006	−0.126	−0.031
Soil	Ln N	0.261^**^	−0.395^**^	−0.509^**^	−0.061	−0.534^**^	−0.122
	Ln P	−0.256^**^	−0.189^*^	−0.112	−0.260^**^	−0.014	−0.299^**^
	Ln N:P	0.440^**^	−0.143	−0.305^**^	0.192^*^	−0.407^**^	0.174^*^

*P < 0.05, **P < 0.01.

**Figure 1:**
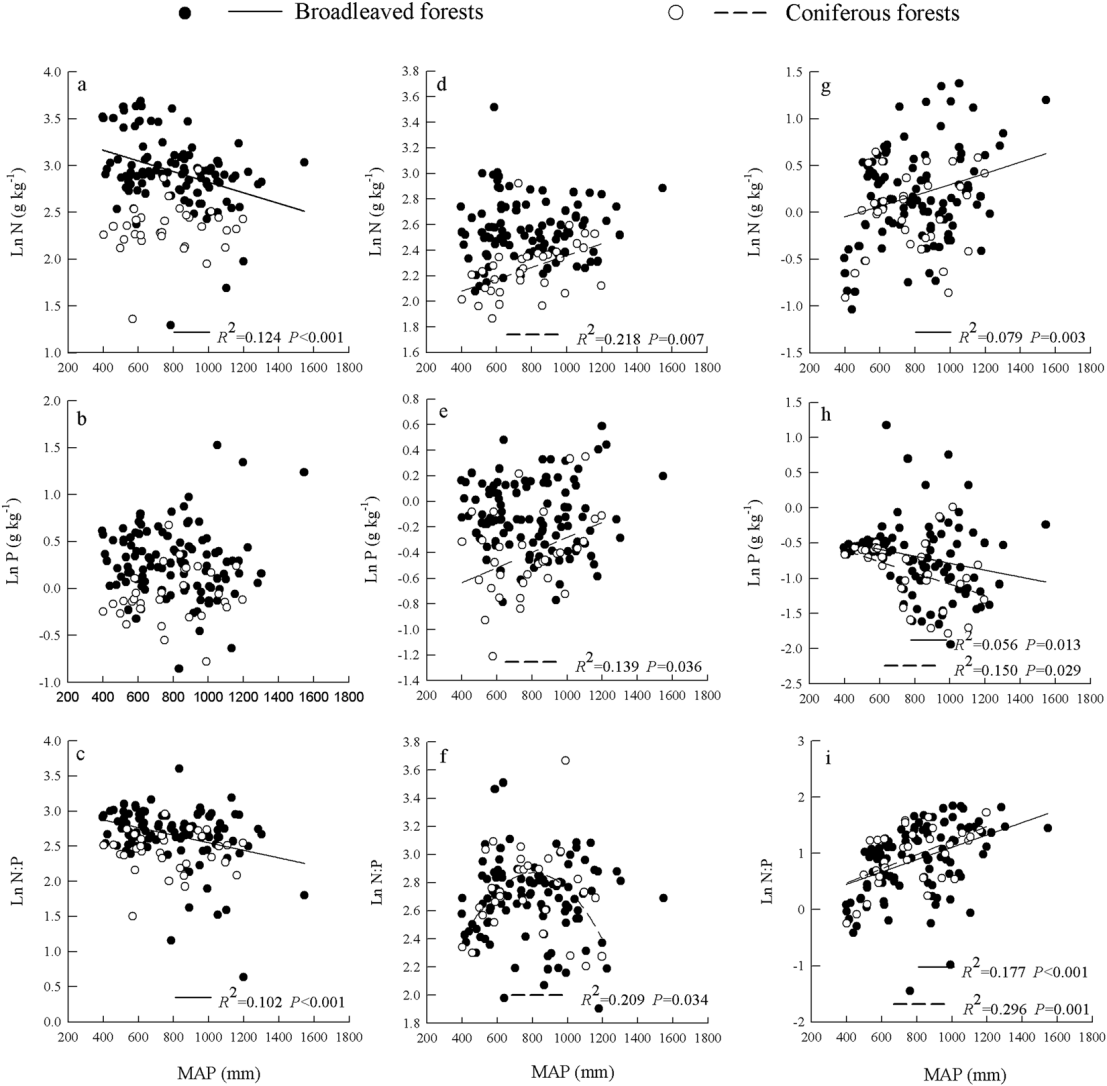
Relationships between MAP and leaf (**a**–**c**), litter (**d**–**f**), and soil (**g**–**i**) stoichiometry for different plant types.

**Figure 2:**
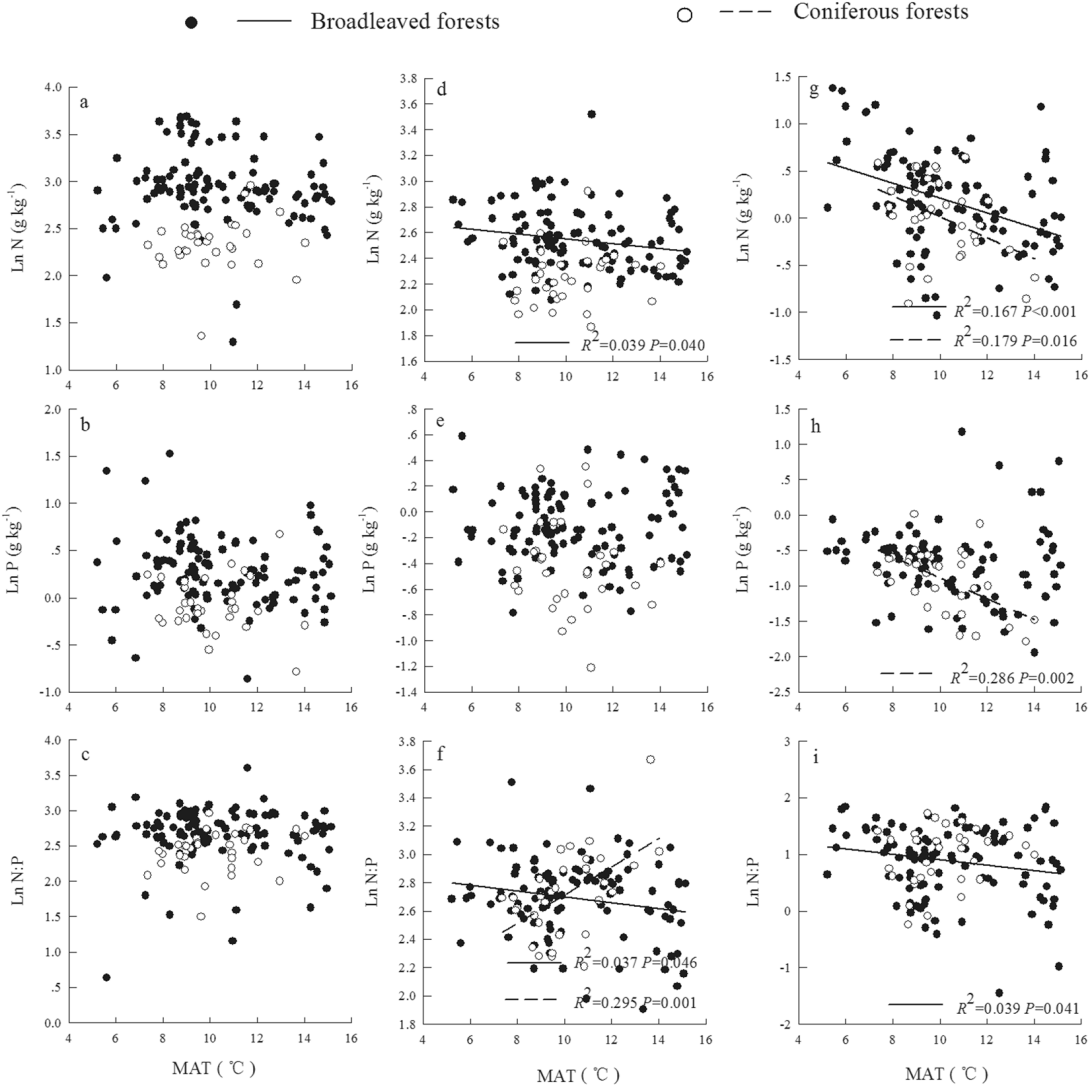
Relationships between MAT and leaf (**a**–**c**), litter (**d**–**f**), and soil (**g**–**i**) stoichiometry for different plant types.

Leaf N and MAP (*R* ^2^ = 0.124, *P* < 0.001) and leaf N: P and MAP (*R* ^2^ = 0.102, *P* < 0.001) were linearly negatively correlated in broadleaved forests, and litter N and MAP (*R* ^2^ = 0.218, *P* = 0.007) and litter P and MAP (*R* ^2^ = 0.139, *P* = 0.036) were linearly positively correlated in coniferous forests. Only a quadratic relationship was found between litter N: P and MAP (R^2^ = 0.209, *P* = 0.034), with a higher N: P observed from 700 mm to 900 mm MAP in coniferous forests. The soil N concentration and N: P in broadleaved forests showed positive linear correlations with MAP (R^2^ = 0.079, *P* = 0.003 and R^2^ = 0.177, *P* < 0.001, respectively), whereas the soil P concentration showed a negative linear correlation with MAP (R^2^ = 0.056, *P* = 0.013). The soil P concentration and N: P had opposite trends with MAP in coniferous forests, where the soil P concentration was linearly positively correlated (R^2^ = 0.150, *P* = 0.029) and N: P was linearly negatively correlated with MAP (R^2^ = 0.296, *P* = 0.001) (Fig. 1). The leaf N and P concentrations and N: P were not significantly correlated with MAT in broadleaved or coniferous forests, whereas the litter N concentration and N: P were linearly negatively correlated with MAT (*R* ^2^ = 0.039, *P* = 0.040 and *R* ^2^ = 0.037, *P* = 0.046, respectively) in broadleaved forests. Litter N: P was linearly and positively correlated with MAT (*R* ^2^ = 0.295, *P* = 0.001) in coniferous forests. The soil N concentration and N: P (*R* ^2^ = 0.167, *P* < 0.001 and *R* ^2^ = 0.039, *P* = 0.041) in broadleaved forests and soil N and P concentrations (*R* ^2^ = 0.179, *P* = 0.016 and *R* ^2^ = 0.286, *P* = 0.002) in coniferous forests were linearly negatively correlated with MAT (Fig. 2).

**Figure 3:**
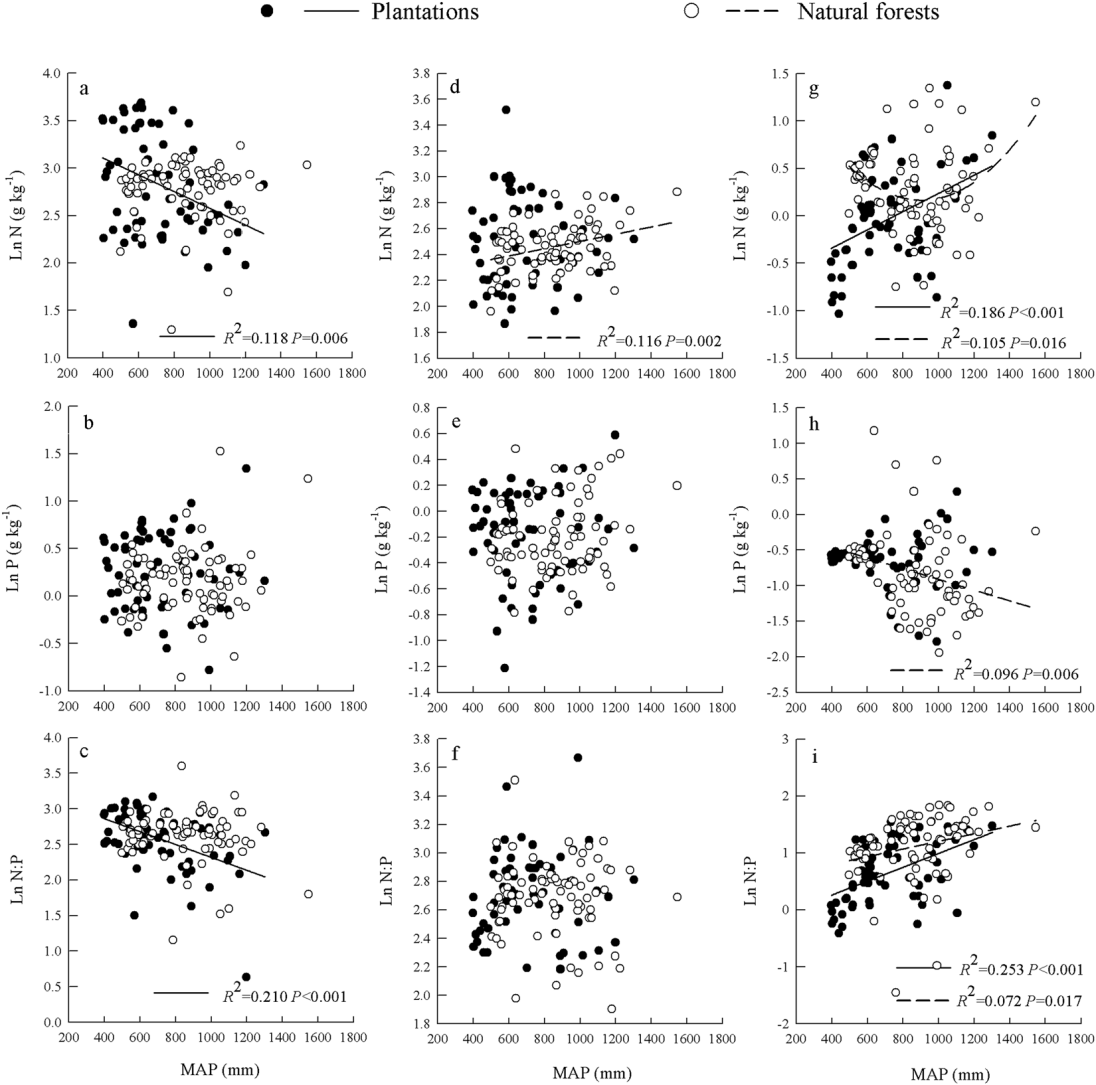
Relationships between MAP and leaf (**a**–**c**), litter (**d**–**f**), and soil (**g**–**i**) stoichiometry for different plant origins.

**Figure 4:**
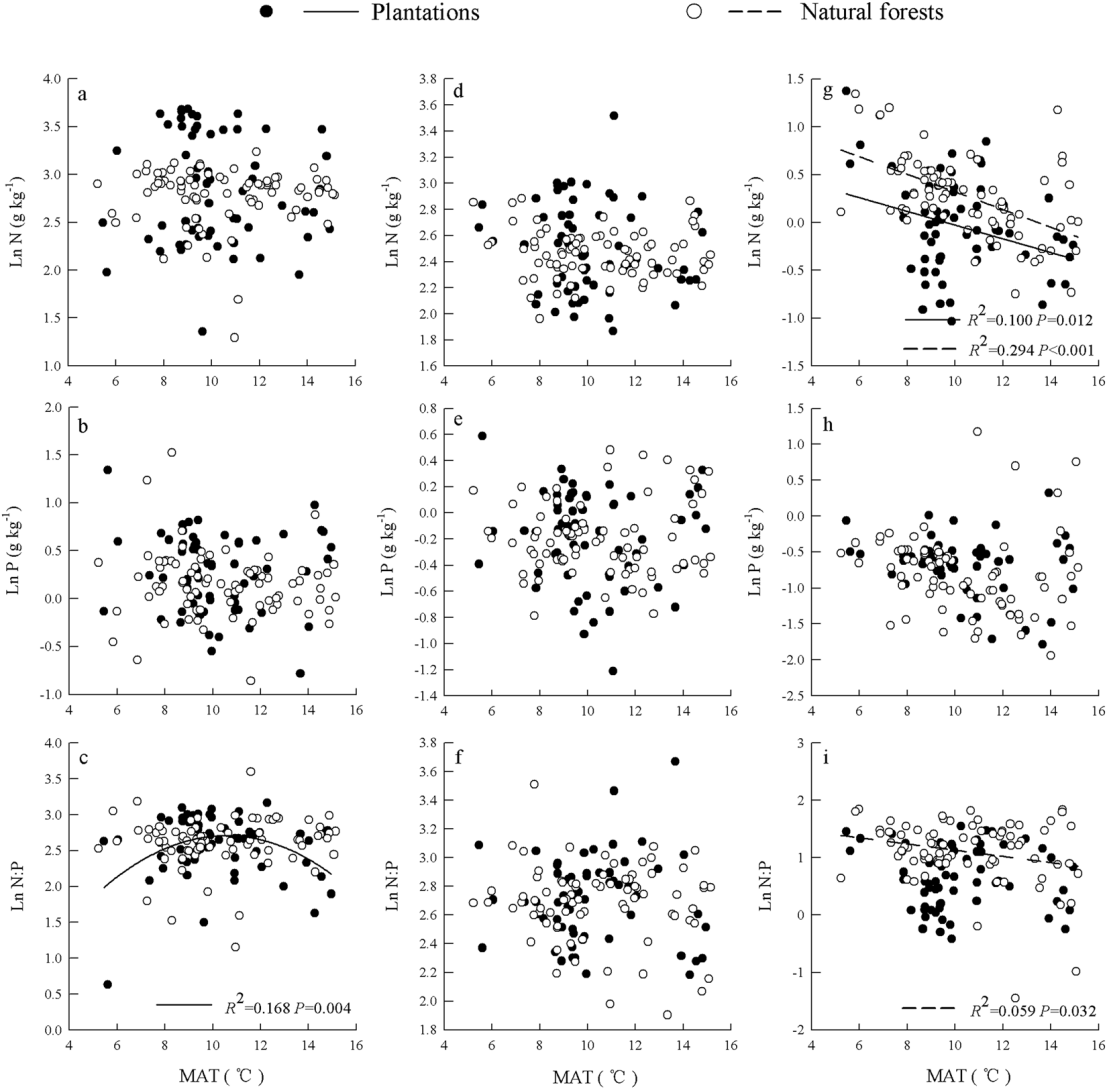
Relationships between MAT and leaf (**a**–**c**), litter (**d**–**f**), and soil (**g**–**i**) stoichiometry for different plant origins.

In plantations, the leaf N concentration and N: P were linearly negatively correlated with MAP (*R* ^2^ = 0.118, *P* = 0.006 and *R* ^2^ = 0.210, *P* < 0.001), and the soil N concentration and N: P were linearly positively correlated with MAP (*R* ^2^ = 0.118, *P* = 0.006 and *R* ^2^ = 0.118, *P* = 0.006). In natural forests, a quadratic correlation was found between soil N and MAP (R^2^ = 0.105, *P* = 0.016), positive linear correlations were found between litter N and MAP (R^2^ = 0.116, *P* = 0.002) and between soil N: P and MAP (R^2^ = 0.072, *P* = 0.017), and a negative linear correlation was found between soil P and MAP (R^2^ = 0.096, *P* = 0.006) (Fig. 3). There was a quadratic correlation was between leaf N: P and MAT (R^2^ = 0.168, *P* = 0.004) and a negative linear correlation between soil N and MAT (R^2^ = 0.100, *P* = 0.012) in plantations. In addition, negative linear correlations were found between soil N and MAT (R^2^ = 0.294, *P* < 0.001) and between soil N: P and MAT (R^2^ = 0.059, *P* = 0.032) in natural forests (Fig. 4).

## Discussion

This study found a broad pattern of relations among leaf, litter, and soil nutrients in the warm temperate forests across northwestern China. Our results revealed that the average leaf C concentration of all 420 samples was 462.9 g kg^−1^, higher than the average of 438.0 g kg^−1^ for 126 samples reported by Zheng and Shangguan^26^ for the Loess Plateau but similar to the global average of 464.0 g kg^−1^ for 398 terrestrial plant species reported by Elser *et al*.^21^ (Supplementary Table S2). The average leaf N and P concentrations in our study were 18.0 and 1.3 g kg^−1^, respectively, lower than those of 41 species (24.1 and 1.6 g kg^−1^, respectively) reported by Zheng and Shangguan^26^ for the Loess Plateau, 554 species (20.2 and 1.4 g kg^−1^, respectively) reported by Han *et al*.^17^ for China, 1251 species (20.1 and 1.7 g kg^−1^, respectively) reported by Reich and Oleksyn^22^ globally, and 398 species (20.6 and 1.9 g kg^−1^, respectively) reported by Elser *et al*.^21^ globally (Supplementary Table S2). Therefore, we analyzed only woody plants and not shrubs and herbs. In addition, the uptake efficiency is higher in herbs than in woody plants^27^, so the leaf N and P concentrations were lower in our study than in the other studies. Leaf N: P was lower than in other studies on the Loess Plateau and China but higher than on a global scale due to the lower soil P concentration in China^28^. The litter N and P concentrations (12.3 and 0.87 g kg^−1^, respectively) were also higher in our study than reported by Kang *et al*.^18^ on a global scale (10.9 and 0.83 g kg^−1^, respectively), but litter N: P was lower (15.45) than previously reported (18.32) (Supplementary Table S2). The soil P concentration (0.54 g kg^−1^) was significantly lower than the global level (2.8 g kg^−1^)^28^, likely due to the strong effects of weathering and erosion on the Loess Plateau and consistent with the generally lower soil P concentration in China than globally^17^. In addition, leaf, litter, and soil P concentrations were more variable than the C and N concentrations in our study (Supplementary Table S1). These findings suggest that the C and N concentrations are more stable and more stoichiometrically homeostatic than the P concentration, consistent with previous studies^7,25,29^. Furthermore, leaf, litter, and soil elements were often significantly correlated individually (Supplementary Table S3), especially the N and P concentrations. Therefore, the variability of N: P is less than the variability of the N and P concentrations, indicating that N: P is relatively stable. The CV was lower for N: P than for the N and P concentrations (Supplementary Table S1), which also confirmed this point.

Some leaf and litter element traits differed significantly between plant morphological types but not between the plant origins (Table 1). The leaf and litter C concentrations were significantly higher and the N and P concentrations were significantly lower in coniferous forests than in broadleaved forests, consistent with other studies^30–32^. Therefore, the leaf and litter C: N and C: P were significantly higher in coniferous forests than in broadleaved forests, indicating that coniferous forests could store more C. The growth rate hypothesis^2,33,34^ states that broadleaved plants (short-lived and fast-growing species) should have higher growth rates than coniferous plants (long-lived and slow-growing species) and should thus be rich in N and P and have higher photosynthetic rates^23,34,35^. The nutrient traits in our study differed little between natural forest and plantation, partly inconsistent with the results of a previous study^36^, perhaps because the natural forests and plantations in our study were located in the areas with similar climatic conditions. However, the soil C and N concentrations and C: P and N: P were higher in natural forest than in plantation, perhaps because of the higher human influence in the plantations.

Some of the traits of the leaf and litter nutrients of all 420 samples were significantly correlated, indicating that the nutrients in the litter were derived from the leaves. The traits of the leaf and litter nutrients, however, were not generally correlated in coniferous forests, indicating that the strategy of growth varied by plant type^37^. The elemental concentrations in leaves are generally positively correlated with the soil’s ability to provide nutrients if plant growth is restricted by one or more elements^38^. Leaf elements were not significantly correlated with soil elements in our study, indicating that plant growth in Shaanxi Province is not restricted by soil nutrients. Leaf N: P is widely used to indicate the availability and limitations of soil nutrients^8^, with an N: P > 16 indicating P limitation and an N: P < 14 indicating N limitation. Either N or P can be limiting at an N: P between 14 and16, and plant growth is co-limited by both N and P. Based on the N: P thresholds used by Koerselman and Meuleman^8^, the coniferous forests in our study were N limited, whereas the broadleaved forests and combined forests were co-limited by both N and P. However, the relationship between leaf and soil nutrients with this standard is not consistent with the status of restricted plant growth in this study: plant growth in Shaanxi Province was not restricted by soil nutrients, perhaps due to different N: P thresholds for different species in different regions used for assessing N and P limitation^39^. The litter and soil nutrient concentrations and ratios were significantly correlated, consistent with a previous study^40^, likely because a large proportion of the nutrients in the litter were released into the soil as litter is a main source of the soil nutrient pool. C: N in the litter is an important indicator of the rates of decomposition and nutrient return, with lower ratios facilitating decomposition^41^. The litter C: N in our study was higher in coniferous than broadleaved forests and all forests combined, indicating that the rate of litter decomposition was lower in coniferous forests, which accounted for the lack of significant correlations between the litter and soil nutrient concentrations and ratios in coniferous forests.

Many studies have focused on broad biogeographic patterns of climatic indices (e.g., MAT or MAP). Reich and Oleksyn^22^ reported that leaf P, and to a lesser degree leaf N, decreased as MAT increased and that leaf N: P increased as MAT increased globally. Reich and Oleksyn^22^ also proposed the temperature-plant physiological hypothesis (TPPH) to account for geographical patterns of variability. The TPPH states that leaf N and P concentrations should decrease with increasing temperature because cold climates may favor high leaf N and P concentrations to compensate for low physiological efficiency at low temperatures. He *et al*.^11^ suggested that plant N and P concentrations should increase monotonically with decreasing temperature, supporting the TPPH. Han *et al*.^17^ reported that the leaf N and P concentrations of 753 plant species in China increased significantly, but leaf N: P did not change significantly, as MAT decreased. However, the leaf N and P concentrations and N: P of the 31 species in Shaanxi Province in our study were not correlated with temperature, except for leaf N: P, which was positively correlated with MAEHT (Table 3). The leaf N and P concentrations and N: P were also weakly correlated with temperature for different plant types and origins (Figs 2 and 4). Our results for the study area in Shaanxi Province were consistent with those reported by Zheng and Shangguan^26^ for the Loess Plateau probably because Shaanxi Province and the Loess Plateau have a small geographical range. In narrow biomes, the leaf nutrient traits of different species fluctuate markedly and the climate varies relatively little, so the spatial patterns of leaf traits modulated by the climate are non-significant^26^. Meanwhile, leaf elements were more significantly correlated with MAP than MAT (Table 3, Figs 1, 2, 3 and 4) in our study. The N and P concentrations and their ratios can indicate the adaptation of plants to local soil conditions^9,42^, and temperature and precipitation may affect leaf elemental concentrations through their influence on the status of soil nutrients^43^. In arid and semi-arid regions, it is a reasonable assumption that precipitation is a more important limiting factor than temperature for vegetation growth. Precipitation is thus more likely to have a greater effect than temperature on plant composition and leaf elemental concentrations in these regions^44^ (Table 3, Figs 1 and 3).

Liu *et al*.^45^ identified two geographically broad trends in the litter N concentration, namely, increasing concentration with increasing MAT and MAP, and Yuan and Chen^24^ found increasing senesced-leaf N concentration and N: P and decreasing senesced-leaf P concentration with increasing MAT and MAP. However, the litter elements in our study were not significantly correlated with either MAP or MAT for the combined forests. Litter elements were more significantly correlated with MAP and less significantly correlated with MAT in coniferous forests than in broadleaved forests (Figs 1 and 2), perhaps because different regions and plant types have different responses to climatic factors. Soil responded more than leaves and litter to climate (Table 3, Figs 1, 2, 3 and 4), possibly because temperature and precipitation affect the leaf elements through their influence on the status of soil nutrients, which can change the composition of plant species^43^. Soils are thus more susceptible to climatic factors.

The soil N and P concentrations were negatively correlated with MAT, but soil N: P remained relatively stable for all forests combined (Table 3), consistent with a study in China by Chen *et al*.^19^, suggesting that the soil N and P concentrations were closely coupled along temperature gradients. By contrast, the soil N concentration and N: P increased and the soil P concentration decreased with MAP in this study. Biological activity in dry lands, such as photosynthesis, atmospheric N fixation, and subsequent microbial mineralization, is primarily driven by water availability. Higher precipitation thus increases the input of soil N by stimulating the activity of the decomposer community but reduces P input by inhibiting mechanical rock weathering^46^. In addition, precipitation in forest ecosystems can increase nutrient leaching from soils instead of increasing nutrient retention, which may deplete N and P and decrease the availability of these elements in the surface soils^12^, potentially accounting for the poor correlations between the leaf and soil nutrients.

## Conclusion

We investigated the ecological stoichiometries of the leaf, litter, and soil nutrients of 31 species at 140 sites in warm temperate forests in northwestern China. The patterns of the elements and theirs ratios differed from those of other studies. The leaf and soil P concentrations were lower but N: P was higher than global values, indicating that Chinese vegetation is universally limited by the availability of P. Precipitation decreases the availability of these elements in surface soils, resulting in leaf and soil nutrients not being significantly correlated. Leaf and litter nutrients as well as litter and soil nutrients were significantly correlated, indicating that litter is a link between plants and soil and demonstrating the coupling among leaf, litter, and soil nutrients. Precipitation had a greater impact than temperature on the plants and soil in Shaanxi Province, perhaps due to the small size of the study area and suggesting that precipitation has a larger effect in arid areas. Our study contributes to the understanding of the cycling of C, N, and P between plants and soil and will assist the prediction of the growth and dynamics of species under climatic or environmental change, especially in fragile regions.

## Materials and methods

### Study site

**Figure 5:**
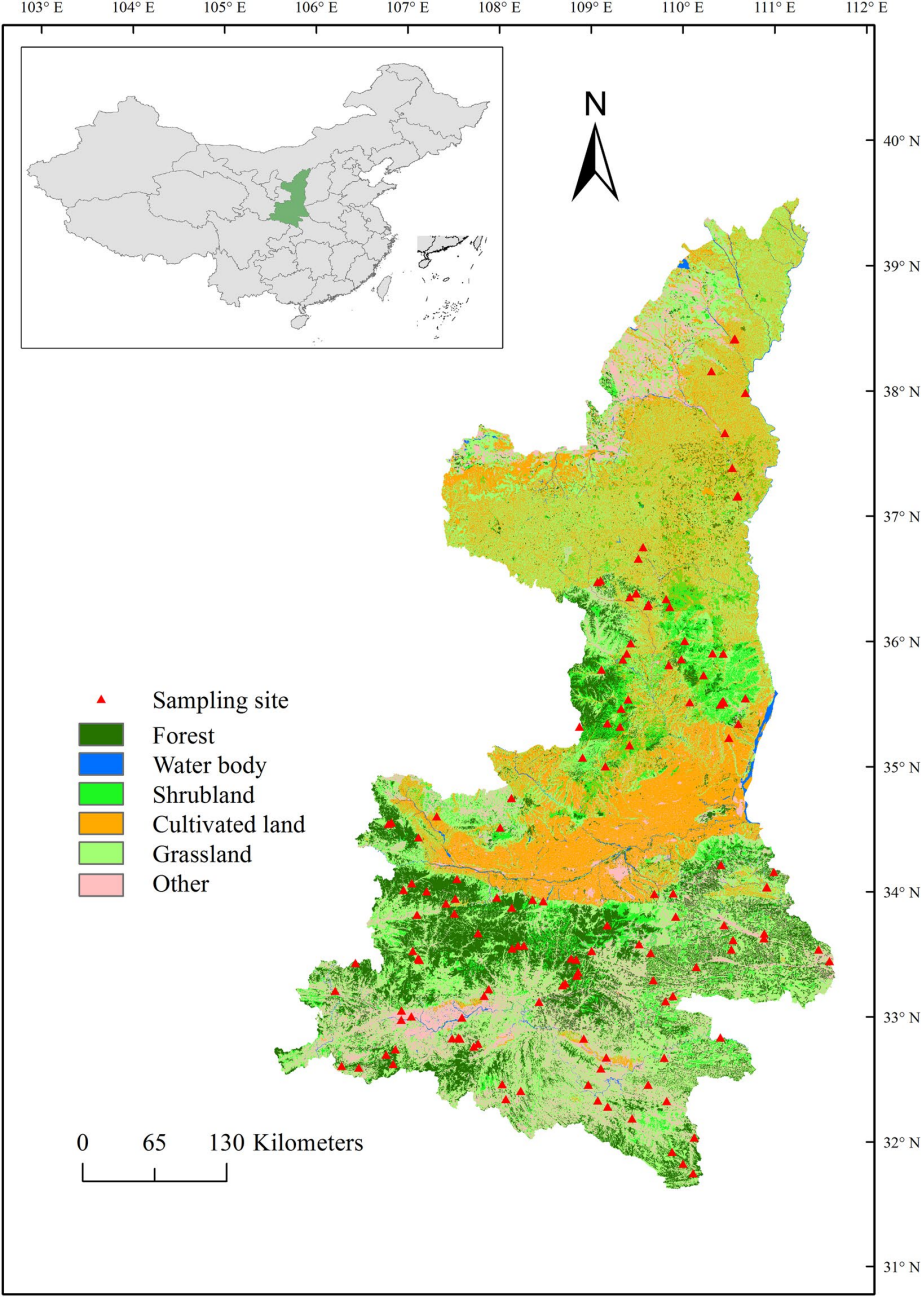
Locations of the sampling sites in the warm temperate forests in northwestern China. Map was generated by ArcMap software (Version 10.2, ESRI, USA).

The study was conducted in Shaanxi Province, northwestern China (31°42′-39°35′N, 105°29′-111°15′E; Fig. 5). This area was selected because of its diverse ecological systems and stable vegetation types and because it is a representative region of vegetation restoration. The climate varies greatly in Shaanxi Province, spanning temperate and warm temperate climatic zones from north to south, with MAT ranging between 8 and 16 °C and MAP ranging between 320 and 1400 mm. Most of the study sites were in the warm temperate zone. With its large area on the Loess Plateau, Shaanxi has initiated many ecological restoration projects to control soil and water loss and for environmental improvement, such as the “Grain for Green” program, a project launched in the 1990s to control soil erosion and increase vegetation cover in degraded lands, which has made important achievements^47^. Forestry resource inventory data indicate that forest coverage in Shaanxi Province was 41.42% in 2009, and the main tree species were *Robinia pseudoacacia*, *Pinus tabulaeformis*, *Cunninghamia lanceolata*, *Cupressus funebris*, *Pinus armandii*, *Larix gmelinii*, *Quercus wutaishanica*, *Quercus acutissima*, *Betula platyphylla*, and *Populus simonii*.

### Leaf, litter, and soil sampling and climate data

We selected 31 dominant species at 140 sampling sites in Shaanxi Province, and three triplicate plots of 20 × 20 m^2^ were studied at each site. The sites were far from grazing activity and anthropogenic disturbances. Samples of leaves, litter, and soil were collected during the growing season (July-August) in 2012. Leaf samples were collected according to the protocol of Cornelissen *et al*.^48^. Fully expanded sunlit leaves were collected from five individual plants in each plot. Leaves from each plot were mixed into one sample for further measurement. Three 1-m^2^ quadrats were randomly placed in each plot to collect the litter, which was then pooled into one sample for analysis. Three soil samples (5 cm in diameter to a depth of 20 cm) were collected in the three litter quadrats and then pooled into one sample for analysis. Totals of 420 leaf samples, 420 litter samples, and 420 soil samples, representing the 31 dominant species, were collected from the 140 sites.

Climatic data were collected from the China Climate Grid Database (resolution of 1 × 1 km) compiled by the Chinese Ecosystem Research Network (www.cnern.org.cn) of the Chinese Academy of Sciences. The database was produced by spatial interpolation and geographic information systems that covered 740 national weather stations in China. These instruments recorded data for each month from 1961 to 2000. The climatic data collected for each site were MAP, MAT, MT_max_, MT_min_, MAEHT, and mean annual extreme low temperature (MAELT).

### Sample measurement

All leaf and litter samples were oven-dried at 80 °C to a constant weight for appropriately 72 h, ground using a ball mill, and sieved through a 0.25-mm mesh screen before chemical analysis. All soil samples were air-dried and ground into a fine powder after removing stones and plant residue. The C concentrations of the leaves, litter, and soil were determined using K_2_Cr_2_O_7_-H_2_SO_4_ wet oxidation, as described by Walkley and Black^49^. The N concentrations were measured using the Kjeldahl method^50^ with H_2_SO_4_ and HClO_4_ for digestion, and the P concentrations were measured using the same digestion solution used for N followed by molybdenum-antimony anti-mix reagent colorimetric analysis^50^. The C, N, and P concentrations of the leaf, litter, and soil samples are expressed as g kg^−1^ on a dry-mass basis, and the C: N: P ratios are reported as mass ratios^51^.

### Data analysis

We classified the tree species into two morphological types, broadleaved and coniferous, and two origins, natural forests and plantations. All variables were reported as means and standard errors (SEs), and the data were ln-transformed to normalize the distributions. One-way ANOVAs were used to compare the characteristics of the leaves, litter, and soil within and between the plant forms. The one-way ANOVA statistics (F, df) are presented in Supplementary Table 4. The relationships among the leaf, litter, and soil nutrient concentrations were estimated using Pearson correlation analyses. Regression analyses and Pearson correlation analyses were used to examine the effects of MAT, MAP, MT_max_, MT_min_, MAEHT, and MAELT on leaf, litter, and soil C, N, and P concentrations and C: N: P ecological stoichiometry across all species. All analyses were conducted with SPSS 19.0 (SPSS Inc., Chicago, IL, USA) at a significance level of *P* = 0.05. Figures were prepared using SigmaPlot 10.0 (Systat Software Inc., Chicago, IL, USA), and the map was generated with ArcMap software (Version 10.2, ESRI, USA).

### Data availability

The datasets generated and/or analyzed during the current study are available from the corresponding author upon reasonable request.

### Ethical approval and informed consent

This article does not contain any studies with human participants or animals performed by any of the authors. Informed consent was obtained from all individual participants included in the study.

## Electronic supplementary material

Supplementary Table s
