# Supplementary material for: The coupling of leaf, litter, and soil nutrients in warm temperate forests in northwestern China
Source: Sci Rep. 2017 Sep 18;7:11754. doi: 10.1038/s41598-017-12199-5 (PMC5603570; doi:10.1038/s41598-017-12199-5)
Supplement: Supplementary file 1 — Supplementary Table s [file 41598_2017_12199_MOESM1_ESM.pdf]

The coupling of leaf, litter, and soil nutrients in warm temperate forests in northwestern China

Guangqi Zhang [1], Ping Zhang [1], Shouzhang Peng [2,3], Yunming Chen [2,3], Yang Cao [2,3*]

[1]College of Forestry, Northwest A＆F University, Yangling 71210, Shaanxi, China

[2]State Key Laboratory of Soil Erosion and Dryland Farming on Loess Plateau, Northwest A＆F University, Yangling 712100, Shaanxi, China

[3]Institute of Soil and Water Conservation, Chinese Academy of Sciences and Ministry of Water Resources, Yangling 712100, Shaanxi, China

*Corresponding author: yang.cao@nwsuaf.edu.cn

Supplementary Table 1 Coefficients of variance for the plant and soil nutrients.

Component	Plant type/origin	C	N	P	C: N	N: P	C: P
Leaf	Broadleaf	0.07	0.37	0.42	0.55	0.31	0.39
	Conifer	0.05	0.26	0.30	0.37	0.27	0.29
	Natural forest	0.09	0.55	0.42	0.67	0.33	0.49
	Plantation	0.07	0.24	0.45	0.51	0.31	0.35
	All	0.08	0.43	0.44	0.59	0.32	0.41
Litter	Broadleaf	0.15	0.27	0.28	0.26	0.28	0.32
	Conifer	0.11	0.24	0.38	0.23	0.35	0.54
	Natural forest	0.19	0.37	0.33	0.43	0.33	0.69
	Plantation	0.13	0.19	0.30	0.21	0.26	0.29
	All	0.16	0.29	0.31	0.33	0.29	0.53
Soil	Broadleaf	0.55	0.53	0.68	0.22	0.51	0.55
	Conifer	0.54	0.40	0.43	0.25	0.44	0.48
	Natural forest	0.60	0.56	0.36	0.28	0.51	0.56
	Plantation	0.50	0.47	0.84	0.20	0.41	0.47
	All	0.55	0.52	0.66	0.24	0.49	0.54

Supplementary Table 2 Comparison of foliar C, N, and P concentrations (g kg^{-1}) and ratios between Shaanxi Province and other regions (mean±standard deviation)

Area	C	N	P	C: N	N: P	C: P	Reference
Shaanxi Province	462.9±35.5	18.0±7.8	1.3±0.5	31.8±18.7	14.3±4.6	411.6±170.5	This study
Loess Plateau	438.0±43.0	24.1±8.5	1.6±0.5	21.2±10.2	15.4±3.9	312.0±135.0	25
China	-	20.2±8.4	1.4±0.9	-	16.3±9.3	-	16
Global	-	20.1±8.7	1.7±1.1	-	13.8±9.4	-	21
Global	464.0±32.1	20.6±12.2	1.9±1.4	22.5±10.6	12.7±6.8	232.0±145.0	20

Supplementary Table 3 Relationships of the C, N, and P contents and their ratios

Component	Nutrient/ratio	C	N	P	C: N	N: P
Leaf	N	-0.490**	1			
	P	-0.367**	0.555**	1		
	C: N	0.609**	-0.989**	-0.565**	1	
	N: P	-0.197*	0.589**	-0.346**	-0.567**	1
	C: P	0.523**	-0.899**	-0.985**	0.631**	0.281**
Litter	N	-0.084	1			
	P	-0.168*	0.556**	1		
	C: N	0.595**	-0.844**	-0.513**	1	
	N: P	0.134	0.263**	-0.606**	-0.157	1
	C: P	0.582**	-0.508**	-0.865**	0.704**	0.585**
Soil	N	0.901**	1			
	P	0.175*	0.223**	1		
	C: N	0.342**	-0.099	-0.089	1	
	N: P	0.580**	0.617**	-0.622**	0.001	1
	C: P	0.662**	0.542**	-0.616**	0.351**	0.936**

*P<0.05, **P<0.01

Supplementary Table 4 One-way ANOVA test statistic values (F, df).

Component	Plant type/origin	Index	C	N	P	C: N	N: P	C: P
Leaf	Broadleaf-Conifer	F	1.175	15.021	5.344	1.904	2.330	0.174
		df	138	138	138	138	138	138
	Natural forest-Plantation	F	19.932	92.262	4.031	11.724	2.133	7.609
		df	138	138	138	138	138	138
Litter	Broadleaf-Conifer	F	2.209	3.371	0.205	4.587	1.303	19.962
		df	138	138	138	138	138	138
	Natural forest-Plantation	F	11.650	27.678	1.901	46.863	2.657	16.889
		df	138	138	138	138	138	138
Soil	Broadleaf-Conifer	F	0.007	3.463	0.699	5.041	0.353	0.177
		df	138	138	138	138	138	138
	Natural forest-Plantation	F	0.001	0.446	4.889	28.694	2.224	1.601
		df	138	138	138	138	138	138
All	Leaf-Litter-Soil	F	104.231	79.744	17.593	47.764	30.006	48.932
		df	417	417	417	417	417	417
